# KCNE1 Constrains the Voltage Sensor of Kv7.1 K^+^ Channels

**DOI:** 10.1371/journal.pone.0001943

**Published:** 2008-04-09

**Authors:** Liora Shamgar, Yoni Haitin, Ilanit Yisharel, Eti Malka, Hella Schottelndreier, Asher Peretz, Yoav Paas, Bernard Attali

**Affiliations:** 1 Department of Physiology and Pharmacology, Sackler Medical School, Tel Aviv University, Tel Aviv, Israel; 2 The Mina and Everard Goodman Faculty of Life Sciences Bar-Ilan University, Ramat-Gan, Israel; Emory University, United States of America

## Abstract

Kv7 potassium channels whose mutations cause cardiovascular and neurological disorders are members of the superfamily of voltage-gated K^+^ channels, comprising a central pore enclosed by four voltage-sensing domains (VSDs) and sharing a homologous S4 sensor sequence. The Kv7.1 pore-forming subunit can interact with various KCNE auxiliary subunits to form K^+^ channels with very different gating behaviors. In an attempt to characterize the nature of the promiscuous gating of Kv7.1 channels, we performed a tryptophan-scanning mutagenesis of the S4 sensor and analyzed the mutation-induced perturbations in gating free energy. Perturbing the gating energetics of Kv7.1 bias most of the mutant channels towards the closed state, while fewer mutations stabilize the open state or the inactivated state. In the absence of auxiliary subunits, mutations of specific S4 residues mimic the gating phenotypes produced by co-assembly of Kv7.1 with either KCNE1 or KCNE3. Many S4 perturbations compromise the ability of KCNE1 to properly regulate Kv7.1 channel gating. The tryptophan-induced packing perturbations and cysteine engineering studies in S4 suggest that KCNE1 lodges at the inter-VSD S4-S1 interface between two adjacent subunits, a strategic location to exert its striking action on Kv7.1 gating functions.

## Introduction

Kv7 channels (Kv7.1-5 or KCNQ1-5) comprise a subfamily of voltage-gated K^+^ channels (Kv), that play important functions in various tissues including epithelia, brain, heart and inner ear organs [1], [2]. Kv7.1 α subunits can interact with various KCNE auxiliary subunits (KCNE1-5), to produce functionally distinct K^+^ currents [3], [4]. Co-assembly of Kv7.1 with KCNE1 generates the *I_KS_* potassium current that is critical for the repolarization of the cardiac action potential [5], [6]. Mutations in either Kv7.1 or KCNE1 genes produce the long QT syndrome (LQT), a genetically heterogeneous cardiovascular disease that is characterized by abnormal ventricular repolarization [1], [7]–[10]. Like all voltage-gated K^+^ channels (Kv), Kv7.1 α subunits are assumed to assemble as tetramers where each monomer consists of six transmembrane segments, including an S5-S6 region encompassing the aqueous pore and a peripheral S1-S4 voltage sensor domain (VSD) [11], [12]. In contrast to many Kv channels, Kv7.1 channels produce striking gating phenotypes when co-expressed with KCNE β subunits. Compared to homomeric Kv7.1 channels, co-assembly of Kv7.1 with KCNE1 produces voltage- and time-dependent K^+^ currents with very slow activation kinetics and a positive shift in the voltage-dependence of activation [5], [6]. When associated with KCNE3 or KCNE2, Kv7.1 yields K^+^ currents that are nearly instantaneous and voltage-independent [13], [14]. Much attention has been paid to the impact of KCNE auxiliary subunits on the pore properties of Kv7.1 α subunits. Several studies suggested that KCNE1 is close to the conduction pathway and may directly interact with the S6 segment of Kv7.1 [15]–[17]. This close interaction between the auxiliary subunit and the pore domain of Kv7.1 has been suggested to underlie the KCNE1 modulation of *I_KS_* channel gating. However, much less is known about the impact of KCNE β subunits on the voltage sensor properties of Kv7.1. Recently, it was shown that the unique S4 charge deficit of Kv7.1 compared to other Kv channels underlies its unique conversion to a leak channel by auxiliary subunits such as KCNE3 [18]. Furthermore, methanethiosulfonate (MTS) accessibility studies indicate that KCNE1 and KCNE3 differentially modulate the voltage sensor of Kv7.1 channels [19], [20]. Here we investigated the nature of the promiscuous gating behavior of Kv7.1 by performing a tryptophan-scanning mutagenesis of the voltage sensor in the absence and presence of KCNE1. In addition, we examined the effect of KCNE1 on disulfide bridge formation in a cysteine engineered in the voltage sensor domain. Our data indicate that tryptophan-induced perturbations of specific S4 residues alter the gating equilibrium distribution by stabilizing Kv7.1 channels in either the open, closed or inactivated states. These S4 perturbations compromise the ability of KCNE1 to properly regulate Kv7.1 channel gating. Along with data of the companion paper, our results also suggest that KCNE1 is strategically located close to S1 and S4 of two adjacent voltage sensing domains.

## Results

### Tryptophan-scanning mutagenesis of Kv7.1 S4 sensor

**Figure 1:**
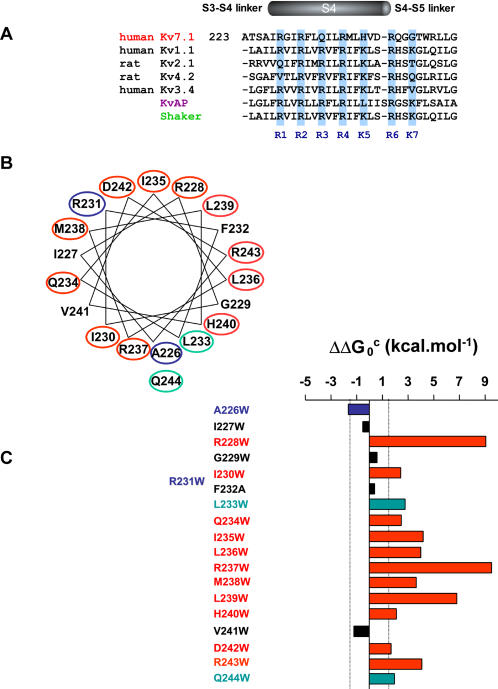
Summary of the tryptophan scan of Kv7.1 S4. (A) Sequence alignment of the S4 segment of human Kv7.1 with various Kv channels. (B) Impact of the perturbations projected onto a helical wheel diagram. The cut-off for significance was ∥ΔΔG_0_^c^∥≥1.5 kcal.mol^−1^. The red, blue and green circled mutated residues shift the gating equilibrium in favor of the closed, open and inactivated state, respectively. (C) Impact of the perturbations expressed as a bar graph along the S4 sequence. The color coding is as in B. The black bars correspond to residues whose perturbation is not significant (∥ΔΔG_0_^c^∥<1.5 kcal.mol^−1^).

Multiple alignment of the S4 segment of KCNQ1 with that of eukaryotic Kv channels and that of the bacterial KvAP reveals significant homology (Figure 1A). However, as recently pointed out [18], compared to the seven basic residues of the *Shaker* and human Kv1.2 sequences, the S4 segment of Kv7.1 contains only 4 basic residues, where R3, K5 and K7 have been replaced by polar amino-acids, Q234, H240 and G246, respectively. Assuming that S4 moves upon membrane depolarization, mutation of a residue to tryptophan is expected to produce a marked gating perturbation if it lies at a protein-protein interface but a weaker alteration if located at a protein-lipid interface. Indeed, tryptophan is assumed to interact favorably with membrane lipids because of its hydrophobic and bulky nature. All mutants were analyzed in CHO cells using the whole-cell patch-clamp technique. The strength of the energetic perturbation produced by each mutation was deduced from conductance-voltage relations, expressed as the free energy difference between wild-type and mutant channels and then corrected for the differences in side chain volume ΔΔG_0_^c^ = ΔG_0_^mut^−ΔG_0_^wt^ (see methods). All gating perturbations were projected onto a helical wheel diagram and plotted as a histogram along the S4 sequence with a cut-off for ∥ΔΔG_0_^c^∥≥1.5 kcal.mol^−1^ (Figure 1B and C and Table 1). Remarkably, all mutants produced functional and K^+^ selective channels, suggesting that the permeation apparatus and the global structure of the channel protein were preserved. Most mutations had a marked impact on channel gating by shifting Kv7.1 gating equilibrium towards the open state, the closed state or the inactivated state, displaying no obvious clustering when projected onto a helical wheel diagram (Figure 1B). The largest group of high-impact residues corresponds to mutations that stabilize the channel in the closed state (Figure 1B and C and Table 1).

**Table 1:** Gating parameters of WT and mutant Kv7.1 channels.

	V_50_ (mV)	z	ΔG_0_ (kcal/mol)	ΔΔG0^c^ (kcal/mol)	I_60_ (pA/pF)
WT Kv7.1 (20)	−29.2±1.3	2.5±0.1	−1.7±0.1		78.9±9.0
A226W (6)	−52.0±3.3*	3.2±0.6	−4.0±0.9	−1.63	37.2±9.6*
I227W (5)	−33.1±3.4	2.4±0.4	−2.0±0.6	−0.52	34.0±6.9*
R228W (8)	11.1±2.9*	1.2±0.1*	0.3±0.1	9.10	40.0±8.3*
G229W (11)	−25.9±1.8	1.1±0.1*	−0.7±0.1	0.59	17.8±4.9*
I230W (8)	−8.8±2.4*	1.8±0.2*	−0.3±0.1	2.44	20.2±4.8*
R231W (8)	NA	NA	NA	NA	101.9±28.4
F232A (8)	−20.9±2.0	2.8±0.2	−1.3±0.2	0.40	51.8±8.5
L233W (13)	−1.9±1.3*	1.1±0.1*	0.1±0.1	2.79	44.2±3.0*
Q234W (6)	2.2±4.0*	1.3±0.1*	0.1±0.1	2.50	31.4±6.2*
I235W (8)	22.1±1.3*	1.3±0.1*	0.7±0.1	4.18	38.7±6.9*
L236W (10)	9.8±1.5*	2.7±0.2	0.6±0.1	4.01	59.9±8.3
R237W (13)	9.7±1.8*	1.6±0.1*	0.4±0.1	9.52	31.3±7.5*
M238W (9)	10.4±2.6*	1.5±0.1*	0.4±0.1	3.66	32.2±4.5*
L239W (5)	39.5±3.6*	2.4±0.3	2.2±0.2	6.80	64.3±5.2
H240W (8)	−11.1±2.7*	1.0±0.1*	−0.3±0.1	2.12	27.8±1.7*
V241W (11)	−51.0±2.4*	2.3±0.3	−2.7±0.4	−1.21	82.7±15.5
D242W (8)	2.9±3.7*	1.0±0.1*	0.1±0.1	1.70	32.1±7.0*
R243W (7)	−16.7±2.2*	2.1±0.1	−0.8±0.1	4.08	87.0±11.4
Q244W (7)	−12.8±4.3*	1.1±0.1*	−0.3±0.1	1.94	29.3±6.1*

V_50_ (half activation voltage) and z (equivalent gating charge) were derived from fitting single Boltzmann function; I_60_ corresponds to the current density measured at +60 mV in pA/pF. ΔG_0_ and ΔΔG_0_^c^ were calculated as described in methods. Data are expressed as mean ± SEM and in parentheses are indicated the number of cells.^*^, p<0.05 compared to WT (two-tailed, Student's unpaired t test). NA, not applicable as R231W mutant is a constitutively open K^+^ leak channel.

### Perturbations stabilizing the open state or destabilizing channel closure

**Figure 2:**
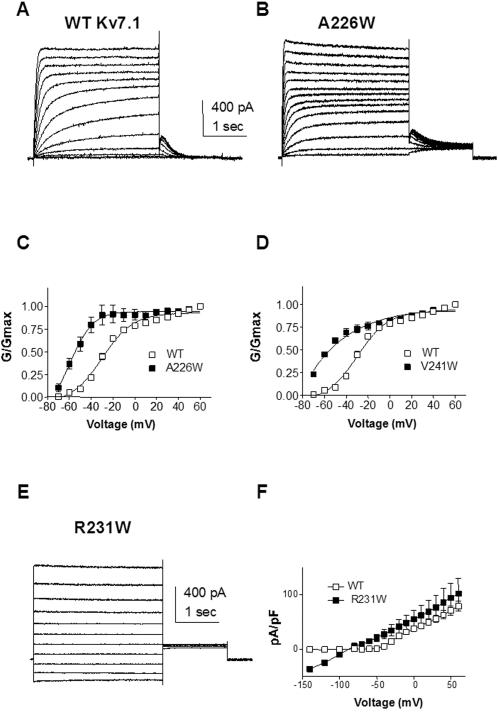
Mutations stabilizing Kv7.1 to the open state. (A) and (B) Representative current traces of WT and A226W, respectively. From a holding potential of −90 mV, the membrane was stepped for 3 s from −70 mV to +60 mV in 10 mV increments and then repolarized for 1.5 s to −60 mV to generate the tail currents. (C) and (D) Normalized conductance was plotted as a function of step voltages, for the mutants (black squares) A226W (n = 6) and V241W (n = 11), respectively, and compared to WT (n = 20) (open squares). The activation curves were fitted using one Boltzmann function. (E) Representative current traces of R231W. Membrane was stepped for 3 s from −140 mV to +60 mV in 20 mV increments and then repolarized for 1.5 s to −60 mV. (F) Current-voltage relations of R231W (n = 8) (black squares) and WT (open squares). Current density (pA/pF) was plotted as a function of step voltages.

CHO cells were held at −90 mV and currents were evoked by voltage steps from −70 mV to +60 mV. A mutation, A226W, located at the S4 N-terminus significantly stabilizes Kv7.1 in the open state. Compared to WT, A226W produces a substantial left-shift of the steady-state activation curve with V_50_ = −52.0±3.3 mV (n = 6) and V_50_ = −29.2±1.3 mV (n = 20) for A226W and WT, respectively (Figure 2A–C and Table 1). The negative shift produced by A226W is accompanied by an increase of the equivalent gating charge z (Table 1), which significantly perturbs the gating energetic towards the open state (ΔΔG_0_^c^ = −1.63 kcal.mol^−1^). Another S4 mutation, V241W, produces a similar left-shift of the activation curve (ΔV_50_ = −21.8 mV). However, the perturbation does not reach significance because of a lower z value (Figure 2D and Table 1). A striking mutation, R231W corresponding to the second basic residue completely disrupts channel closure by producing instantaneous and voltage-independent K^+^ currents (Figure 2E and F). Compared to WT, R231W channels totally lose their voltage dependence, a feature characteristic of the phenotype produced by co-expressing WT Kv7.1 with KCNE3 or KCNE2 β subunits [13], [14]. Stepping the membrane voltage from −140 mV to +60 mV, produces constitutively open channels whose currents reverse at the expected reversal potential for K^+^ (about −85 mV, Figure 2F).

### Perturbations stabilizing the closed state or the inactivated state

**Figure 3:**
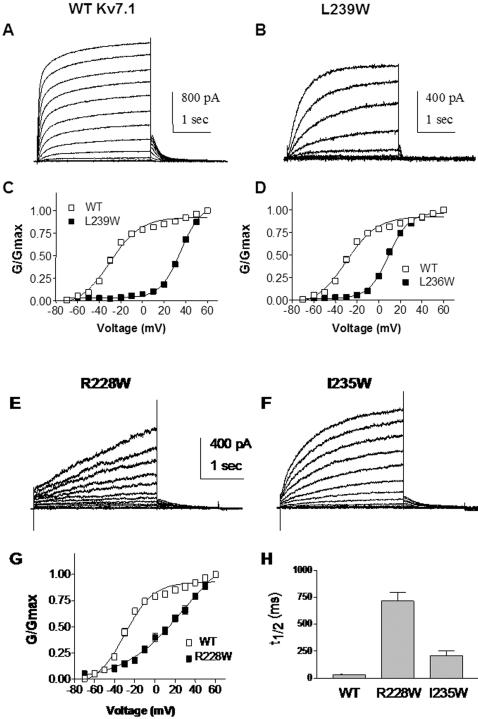
Mutations stabilizing Kv7.1 to the closed state. (A) and (B) Representative current traces of WT and L239W, respectively, recorded as in Figure 2 A. (C) and (D) Normalized conductance of the mutants (black squares) L239W (n = 5) and L236W (n = 10), respectively, compared to WT (open squares). (E) and (F) Representative current traces of R228W and I235W, respectively, recorded as in Figure 2 A. (G) Normalized conductance of R228W (n = 8) (black squares), compared to WT (open squares). (H) The time t_1/2_ needed to reach half-maximal current amplitude was determined for R228W, I235W and WT (n = 8–20).

The vast majority of S4 mutants exhibit relatively large energetic perturbations that shift the gating equilibrium in favor of the closed state, with positive ΔΔG_0_^c^ values ranging from 1.7 to 9.52 kcal.mol^−1^ (Figure 1C and Table 1). Some mutations such as L239W, I235W, R228W, L236W or R237W generate profound positive shifts in the voltage dependence of channel activation, going from ΔV_50_ = +38.9 mV to ΔV_50_ = +68.7 mV (Figure 3 and Table 1). In many of these mutants, there is a significant reduction of the gating charge z, compared to WT (Table 1). The perturbation at some residues like I235 or R228 not only produces a marked right-shift of the conductance-voltage relation but also considerably slows down the activation kinetics (Figure 3E–H). In fact, the gating behavior of I235W and R228W mutants is similar of that produced by co-expressing of WT Kv7.1 with KCNE1. In other mutants like L239W, I230W or M238W, the positive shift in the voltage dependence of activation is accompanied by a marked acceleration of channel deactivation with τ_deact_ = 40±3 ms (n = 5), τ_deact_ = 80±6 ms (n = 8) and τ_deact_ = 89±12 ms (n = 9), respectively, in comparison to WT Kv7.1 (τ_deact_ = 372±40 ms, n = 20) (Figure 3A and B).

**Figure 4:**
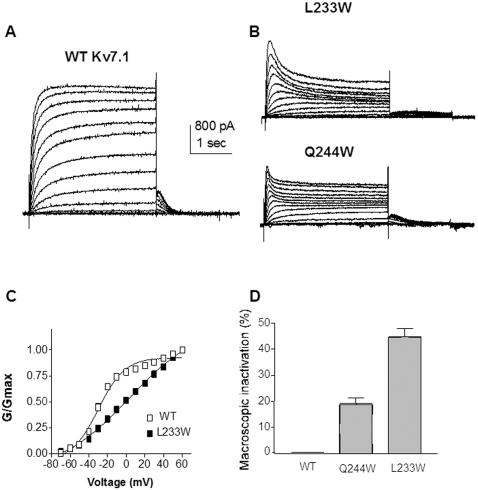
Mutations stabilizing KCNQ1 towards the inactivated state. (A) and (B) Representative current traces of WT and L233W and Q244W, respectively, recorded as in Figure 2 A. (C) Normalized conductance of L233W (n = 13) (black squares), compared to WT (open squares). (D) Percent of macroscopic inactivation of WT, Q244W and L233W (n = 7–20) as measured by the ratio between the sustained and the peak current amplitudes.

Reflecting the multiple facets of perturbations produced by tryptophan substitutions in Kv7.1 S4, two mutations stabilize the inactivated state. Mutants L233W and Q244W, which shift the gating equilibrium towards the closed state, also stabilize the channel in the inactivated state and in fact exhibit a voltage-dependent macroscopic inactivation (Figure 4A–D). As measured by the ratio between the sustained and the peak current amplitudes, the macroscopic inactivation is 45±3% (n = 13) and 19±2% (n = 7) for L233W and Q244W, respectively (Figure 4B and D).

### KCNE1 function is disrupted by tryptophan-induced perturbations in S4

**Figure 5:**
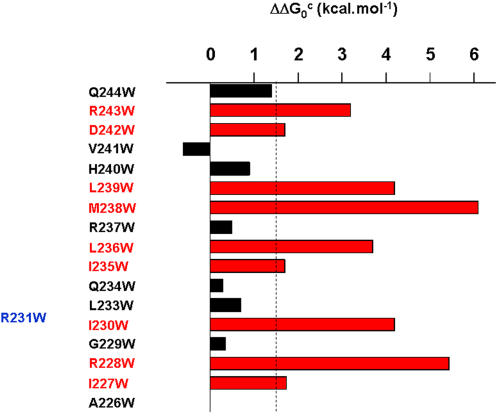
Summary of the tryptophan scan of Kv7.1 S4 in the presence of KCNE1. The cut-off for significance was ∥ΔΔG_0_^c^∥≥1.5 kcal.mol^−1^. The red and blue bars of the mutated residues shift the gating equilibrium in favor of the closed and open state, respectively. The black bars correspond to residues whose perturbation is not significant (∥ΔΔG_0_^c^∥<1.5 kcal.mol^−1^).

**Figure 6:**
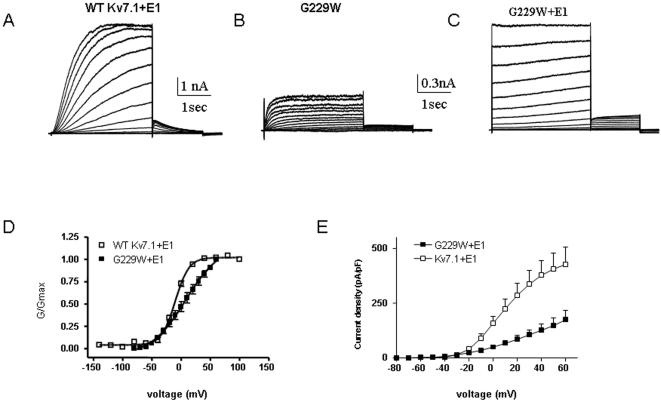
Effect of KCNE1 co-expression with mutant G229W. Representative current traces of WT I_KS_ (A) and mutant G229W expressed without (B) or with KCNE1 (C). From a holding potential of −90 mV, the membrane was stepped for 3 s from −70 mV to +60 mV in 10 mV increments and then repolarized for 1.5 s to −60 mV to generate the tail currents. Conductance-voltage relations (D) and current-voltage relations (E) of WT Kv7.1 and mutant G229W co-expressed with KCNE1.

**Figure 7:**
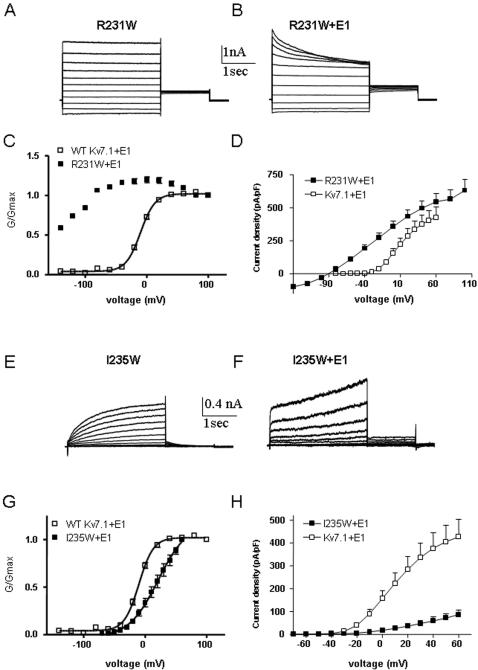
Effect of KCNE1 co-expression with mutant R231W and I235W. Representative current traces of mutant R231W expressed without (A) or with KCNE1 (B). Conductance-voltage relations (C) and current-voltage relations (D) of WT Kv7.1 and mutant R231W co-expressed with KCNE1. Representative current traces of mutant I235W expressed without (E) or with KCNE1 (F). Conductance-voltage relations (G) and current-voltage relations (H) of WT Kv7.1 and mutant I235W co-expressed with KCNE1.

**Figure 8:**
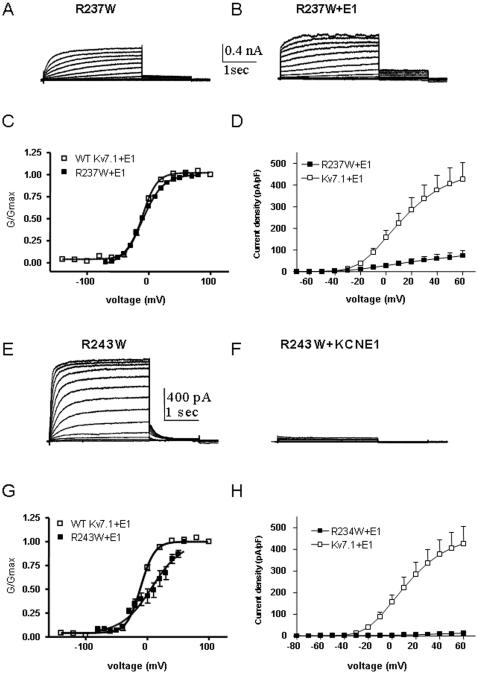
Effect of KCNE1 co-expression with mutant R237W and R243W. Representative current traces of mutant R237W expressed without (A) or with KCNE1 (B). Conductance-voltage relations (C) and current-voltage relations (D) of WT Kv7.1 and mutant R237W co-expressed with KCNE1. Representative current traces of mutant R243W expressed without (E) or with KCNE1 (F). Conductance-voltage relations (G) and current-voltage relations (H) of WT Kv7.1 and mutant R243W co-expressed with KCNE1.

To examine the impact of KCNE1 on tryptophan-induced perturbation of S4 residues, all mutants were co-expressed in the presence of WT KCNE1 in CHO cells. For several S4 mutants, the presence of KCNE1 does not considerably alter the energetics of the tryptophan-induced perturbation (Figure 5 and Table 2). For example, KCNE1 does not noticeably affect the trend of mutants like R228W, I230W, I235W, L236W, M238 or L239W which exhibit large energetic perturbations that shift the gating equilibrium in favor of the closed state, with positive ΔΔG_0_^c^ values (Figure 5 and Table 2). However, there is a series of S4 residues for which the ability of KCNE1 to normally modulate channel gating is severely impaired. In WT *I_KS_*, KCNE1 produces a right-shift in the voltage-dependence of activation, slows down the activation kinetics and suppresses inactivation [5], [6]. At the S4 N-terminus, mutant G229W generates a current whose gating properties are comparable to those of WT Kv7.1 (Figure 6). However, KCNE1 is unable to reproduce the characteristic slow *I_KS_* current. Instead, an instantaneous non-deactivating current is generated (Figure 6C). A striking mutation, R231W corresponding to the second arginine in S4 completely disrupts channel closure, producing instantaneous and voltage-independent leak K^+^ currents whose modulation by KCNE1 is disrupted. KCNE1 is unable to slow down the activation kinetics and produces a time- and voltage-dependent K^+^ current (Figure 7A–D). At the C-terminal half of S4, two mutations I235W and R237W generate currents with right-shifts in their activation curves and slower activation kinetics than WT Kv7.1. Again, these S4 perturbations prevent KCNE1 to properly regulate channel activity (Figures 7E–H and 8A–D). Instead, mutant channels open instantaneously and do not close normally. For mutant R237W, KCNE1 markedly lowers the positive ΔΔG_0_^c^ value obtained when R237W is expressed alone (from ΔΔG_0_^c^ = 9.52 kcal.mol^−1^ to ΔΔG_0_^c^ = 0.50 kcal.mol^−1^, Figures 1 and 5, Tables 1 and 2). At the C-terminal end of S4, the mutant R243W produces a current whose gating characteristics are similar to those of WT Kv7.1 (Figure 8E–H). However, co-expression with KCNE1 produces a dramatic inhibition (99% inhibition) of the current, though the channel protein is targeted to the plasma membrane (not shown). The perturbation at R243W remarkably hampers the ability of KCNE1 to properly modulate channel activity. Altogether, these data indicate that perturbations applied to several residues in the S4 helix markedly compromise the ability of KCNE1 to normally regulate Kv7.1 channel gating.

**Table 2:** Gating parameters of WT and mutant Kv7.1 channels expressed in the presence of WT KCNE1.

	V_50_ (mV)	z	ΔG_0_ (kcal/mol)	ΔΔG0^c^ (kcal/mol)	I_60_ (pA/pF)
WT Kv7.1+KCNE1 (16)	−10.1±0.8	2.2±0.1	−0.5±0.1		426±78
A226W+KCNE1 (11)	−12.7±0.6	1.6±0.1	−0.5±0.1	0	551±73
I227W+KCNE1 (13)	11.2±0.3*	1.9±0.2	0.5±0.1	1.74	406±98
R228W+KCNE1 (9)	22.2±0.7*	1.4±0.2*	0.7±0.2	5.44	235±67*
G229W+KCNE1 (10)	8±2.2*	0.8±0.1*	0.1±0.1	0.35	173±44*
I230W+KCNE1 (12)	60.2±0.8*	1.4±0.2	1.9±0.2	4.2	114±22*
R231W+KCNE1 (8)	NA	NA	NA	NA	546±62
F232A+KCNE1	ND	ND	ND	ND	ND
L233W+KCNE1 (11)	−2.3±0.3*	2.0±0.2	−0.1±0.1	0.7	320±88
Q234W+KCNE1 (11)	−7.5±0.9	1.8±0.1	−0.3±0.1	0.3	338±57
I235W+KCNE1 (8)	21.4±2.5*	1.1±0.1*	0.5±0.1	1.7	87±21*
L236W+KCNE1 (8)	61.8±2.4*	1.1±0.1*	1.6±0.2	3.7	56±12*
R237W+KCNE1 (10)	−10.2±0.7	1.5±0.1	−0.4±0.1	0.5	74±22*
M238W+KCNE1 (12)	61.3±2.0*	2.1±0.2	3.0±0.2	6.1	76±14*
L239W+KCNE1 (7)	40.1±0.5*	2.1±0.2	1.9±0.2	4.2	152±29*
H240W+KCNE1 (12)	3.5±0.6*	1.6±0.1*	0.1±0.1	0.9	358±74
V241W+KCNE1 (13)	−26.2±0.6*	1.6±0.1*	−1.0±0.1	−0.6	403±53
D242W+KCNE1 (10)	55.1±7.6*	1.0±0.1*	1.3±0.1	1.7	17±6*
R243W+KCNE1 (6)	7.5±3.8*	1.0±0.2*	0.2±0.1	3.2	13±5*
Q244W+KCNE1 (10)	16.8±2.0*	1.4±0.1*	0.5±0.1	1.4	210±45*

V_50_ (half activation voltage) and z (equivalent gating charge) were derived from fitting single Boltzmann function; I_60_ corresponds to the current density measured at +60 mV in pA/pF. ΔG_0_ and ΔΔG_0_^c^ were calculated as described in methods. Data are expressed as mean ± SEM and in parentheses are indicated the number of cells.^*^, p<0.05 compared to WT (two-tailed, Student's unpaired t test). ND, not determined; NA, not applicable as R231W mutant is a constitutively open K^+^ leak channel.

### KCNE1 disturbs the open state stabilization effect of Cu-Phen on S4 mutant R228C

**Figure 9:**
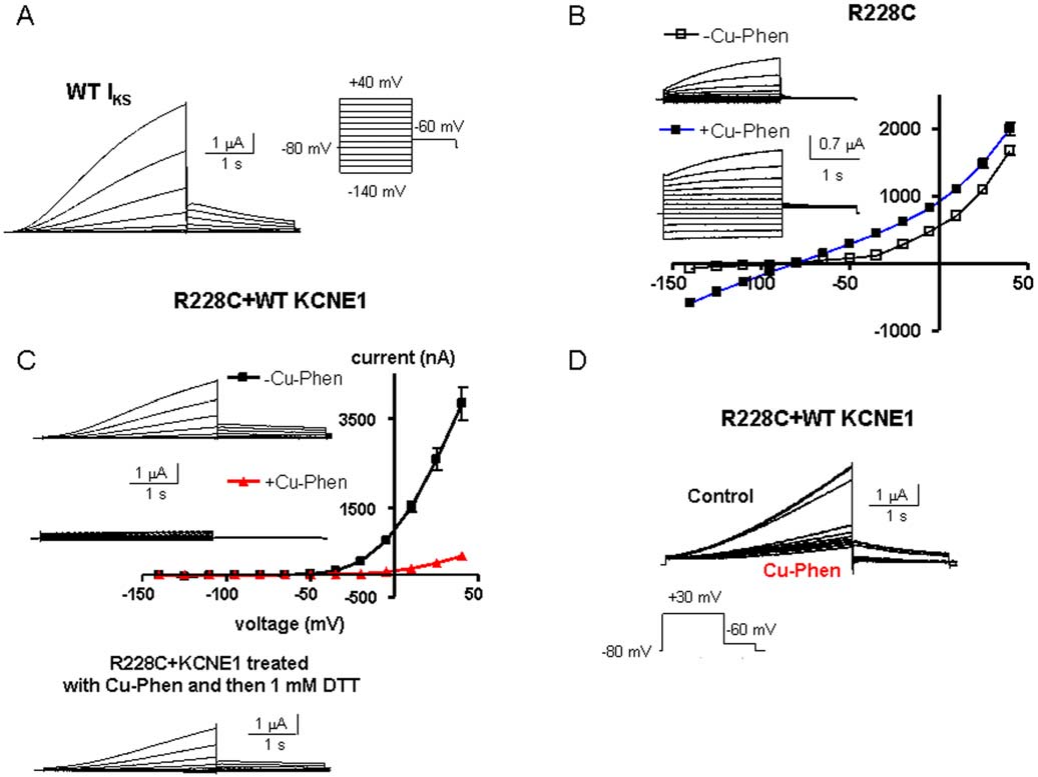
Impact of KCNE1 expression on WT Kv7.1 and mutant R228C. (A) Representative trace of WT Kv7.1 coexpressed with WT KCNE1. (B) Effects of external Cu-Phen on mutant R228C. Oocytes were bathed in ND96 in the absence and presence of 100 µM Cu-Phen. Shown are representative traces and current-voltage relations were determined as indicated. (C) Shown are representative traces and current-voltage relations of R228C+WT KCNE1 channels, when oocytes were bathed with ND96 in the absence of presence of 100 µM Cu-Phen. Also shown, is the reversal by DTT of the current decrease produced by Cu-Phen. (D) Representative traces of R228C+WT KCNE1 channels, when oocytes were bathed with ND96 containing 100 µM Cu-Phen. Currents were evoked by a train of step depolarization to +30 mV. Similar results have been obtained in 5 other cells.

In the companion paper using the *Xenopus* oocyte expression system, we showed that the cysteine mutant R228C in S4 is stabilized in the open state by external Cd^2+^ (100 µM) or copper-phenanthroline (100 µM, Cu-Phen; 1∶3 ratio). We found that upon external exposure of the oocyte to Cd^2+^ (100 µM) or Cu-Phen (100 µM), the time- and voltage-dependent outwardly-rectifying shape of the R228C current is switched to a nearly linear leak character with currents devoid of time and voltage dependence (Figure 9B). In fact, we found that a disulfide bridge could form between R228C in S4 and C136 in S1 from an adjacent VSD, which stabilizes the channel open state. Hence, we checked the impact of WT KCNE1 on mutant R228C channels when treated with Cu-Phen. Compared to WT *I_KS_* (Figure 9A), R228C+KCNE1 produced a very similar slowly activating voltage-dependent K^+^ current (Figure 9C). Cu-Phen (100 µM) markedly decreased the current amplitude of R228C+KCNE1, contrary to its activating effect on R228C expressed alone. Figure 9D illustrates the rapid suppression of the current when the oocytes were exposed to Cu-Phen and stimulated by a train protocol where the membrane potential was stepped every 30 s to +30 mV. The IV relation indicates that Cu-Phen depressed the current amplitude of R228C+KCNE1 at all voltages (at +40 mV, ∼90% inhibition, n = 10, p<0.001). The inhibition of the current amplitude by Cu-Phen was reversed only following incubation with ND96 containing 1 mM DTT (Figure 9C, lower panel). These data suggest that KCNE1 disrupts the open state constraint between R228C in S4 and C136 in S1, in such a way that in the presence of Cu-Phen it favors a different disulfide bridge formation that stabilizes the closed state.

## Discussion

In spite of a relatively high degree of homology of the S4 sequence among Kv channels, Kv7.1 channels are unique in their gating behavior. When expressed as homomeric α subunits, Kv7.1 elicits a typical delayed-rectifier voltage-dependent K^+^ current. However, Kv7.1 can interact with various KCNE β subunits to form K^+^ channels with very different gating behaviors going from time- and voltage-independent K^+^ currents to very slow and high-threshold activation voltage-dependent K^+^ currents [3], [4]. The present study highlights three main features. (a) A set of striking gating behaviors is generated by mutating residues along the S4 voltage sensor. (b) Perturbing the S4 energetics of Kv7.1 mimics many of the gating phenotypes induced by co-assembly of WT Kv7.1 with KCNE auxiliary subunits. (c) Packing perturbation and cysteine mutation studies in S4 suggest that KCNE1 lodges at the inter-VSD S4-S1 interface between two adjacent subunits and thereby affects Kv7.1 sensor functions.

Our data suggest that there are significant differences in voltage sensing between Kv7.1 and other Kv channels. While many Kv7.1 tryptophan mutations stabilized the closed state, only two mutants A226W and R231W shifted the gating equilibrium towards the open state. This feature contrasts with that obtained from an alanine scan in Kv2.1 where five mutated residues in the S4 N-terminus stabilize the open state [21]. It is also different from *Shaker* channels where four hydrophobic and charge neutralization mutants in S4 stabilize the open state [22], [23]. Another remarkable difference resides in the R231W mutation which completely prevents the closure of Kv7.1 channels, a feature also described in a recent study [18]. R231W locks the channel in the open state which circumvents the need of voltage-dependent motions of S4. Mutation of R231 does more than simply changing the gating charge; it probably alters the stability of the packing structure of S4 and its interface with other parts of the channel protein. In the model of the Kv7.1 channel open state, mutant R231W interacts with a hydrophobic pocked located at the upper S5 helix that is formed by residues Y278, F279 and L282. The lack of time-dependence in R231W currents further suggests a disruption of the need of voltage sensing for channel activation, a feature similar to that obtained when WT Kv7.1 is co-expressed with KCNE3 or KCNE2 [13], [14]. These results indicate that the interactions between Kv7.1 and KCNE β subunits may stabilize distinct allosteric states by allowing the channel to adopt specific gating modes at lower energetic cost.

**Figure 10:**
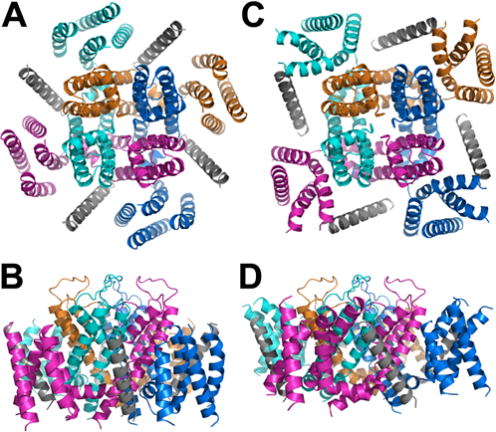
Models corresponding to the three-dimensional structure of Kv1.7 K+ channel and its auxiliary subunit KCNE1. (A) and (B), the closed state in top and side views, respectively. (C) and (D), the open state in top and side views, respectively. Ribbon diagrams of four differently colored identical subunits with the membrane embedded helical segment of KCNE1 in grey.

Our data suggest that KCNE1 disrupts the open state constraint at the inter-VSD interface between S1 and S4. Hence, in the presence of Cu-Phen KCNE1 favors a disulfide bridge formation that stabilizes the Kv7.1 closed state, possibly by allowing an intra-VSD bridge between C136 and R228C. Indeed, in the closed state model R228 points close to C136 of the same VSD (see companion paper). We posit that KCNE1 lodges between S1 and S4 segments of two adjacent subunits. Figure 10 illustrates this putative location which is in line with a recent study showing that in the open state, residue E43 at the external boundary of KCNE1 transmembrane segment is close enough to residue A226 at the S4 N-terminus of Kv7.1 to form a disulfide bond when mutated to cysteines [19]. This data supports the hypothesis that KCNE1 directly interacts with the S4 segment to regulate voltage sensing function. In this presumed topology, residue Y65 at the bottom of the KCNE1 membrane-spanning helix is confined close to residue R259 at the S4-S5 linker, which is a strategic location for modulating channel gating (Figure 10). KCNE1 could thus modulate Kv7.1 gating by impinging on two crucial region of the VSD: (1) the interface between S1 (C136) and S4 (R228) of adjacent subunits and (2) the S4-S5 linker that couples the VSD motion to channel gate. By doing so, KCNE1 may produce the slow, sigmoidal *I_KS_* activation kinetics and the right-shift in voltage-dependence of channel activation. The slow, voltage-dependent gating mode displayed by co-expression of Kv7.1 with KCNE1 may result either from a slowed movement of the constrained VSD or from a delayed coupling between S4 motion and opening of the activation gate. Along this line, two recent studies using accessibility of engineered cysteines at the Kv7.1 S4 to MTS reagents showed that residues A226, R228, G229 and I230 were modified by MTSET only upon membrane depolarization and that their modification rate was markedly slowed down by KCNE1 [19], [20]. In contrast, in the presence of KCNE3 these residues were accessible to MTSET independent of voltage, a feature consistent with KCNE3 shifting the voltage sensor equilibrium to favor the active state at hyperpolarizing potentials [19], [20]. However, it remains to be determined whether KCNE1 slows the motion of the VSD or/and that of the activation gate.

By which mechanisms KCNE1 could alter this inter-VSD interface between S1 and S4? We suggest that KCNE1 affects the packing of S4 residues. The results of the tryptophan scan perturbation identified a series of S4 residues for which the ability of KCNE1 to properly regulate Kv7.1 channel gating has been severely impaired. Thus, when the normal packing of S4 is disturbed by bulky tryptophan substitutions, KCNE1 is unable to reproduce the characteristic slow *I_KS_* current. Instead, mutant channels open instantaneously and do not close normally in mutants G229W, R231W, I235W and R237W. For example, the mutant G229W at the S4 N-terminus may be in close proximity to residue E43 at the N-terminal boundary of the KCNE1 transmembrane segment and therefore stabilizes the channel open state conformation. In contrast, a perturbation in the S4 C-terminus, R243W, dramatically prevents KCNE1 to open the mutant channel. Residue R243 resides at the boundary of the S4 helix and the S4-S5 linker, a probably critical interface for the functional interaction between Kv7.1 and KCNE1. When expressed alone, R243W has an apparently normal sensor function and expresses functional K^+^ currents. However, co-expression of R243W and KCNE1 dramatically suppresses the current, suggesting that KCNE1 prevents mutant channel opening by disrupting either S4 movement or the coupling between S4 and the activation gate. It is possible that residue R243 may interact with the cytoplasmic boundary of the KCNE1 transmembrane segment. The importance of residue R243 for proper KCNE1 function is underscored by the existence of naturally occurring long QT mutations (R243C, R243H) leading to profound perturbations in channel gating [24].

The role of the S1 C-terminus in proper KCNE1 function and its importance in channel gating is underscored by the recently described familial atrial fibrillation mutation in S140G. This Kv7.1 mutation in the presence of KCNE1 generates large instantaneous, voltage-independent leak K^+^ currents and leads to a gain of function [25]. Thus, a location of KCNE1 between two adjacent subunits at the inter-VSD interface S4-S1 may well account for its impact on Kv7.1 channel gating.

In all, this study indicates that despite the high degree of conservation of the S4 sequence among Kv channels, Kv7.1 channels exhibit a significant uniqueness. It also suggests that for exerting its striking effect on channel gating, KCNE1 needs to be strategically located close to S1 and S4 of two adjacent VSDs, thereby constraining sensor movement.

## Materials and Methods

### 

#### Channel expression and two-electrode voltage-clamp recording in Xenopus oocytes

Female *Xenopus* Laevis frogs were purchased from Xenopus 1 (Dexter, Michigan, USA). The procedures followed for surgery and maintenance of frogs were approved by the animal research ethics committee of Tel Aviv University and in accordance with the Guide for the Care and Use of Laboratory Animals (1996. National Academy of Sciences, Washington D.C.). Frogs were anaesthetized with 0.15% tricaine (Sigma). Pieces of the ovary were surgically removed and digested with 1 mg/ml collagenase (type IA, Sigma) in Ca^2+^-free ND96 for about one hour, to remove follicular cells. Stage V and VI oocytes were selected for cRNA injection and maintained at 18°C in ND96 (in mM: 96 NaCl, 2 KCl, 1.8 mM CaCl_2_, 1 MgCl2 and 5 HEPES titrated to pH = 7.5 with NaOH), supplemented with 1 mM pyruvate and 50 µg/ml gentamycin. The human Kv7.1 cDNA (in pGEM vector) was linearized by Not1. This vector served also as a template to generate the Kv7.1 mutants, using site-directed mutagenesis performed by the QuikChange (Stratagene) method. All mutant sequences were verified by DNA sequencing. Capped complementary RNA was transcribed by the T7 RNA polymerase, using the mMessage mMachine transcription kit (Ambion Corp). The cRNA size and integrity was confirmed by formaldehyde-agarose gel electrophoresis. Expression of WT and Kv7.1 mutants was performed by injecting 40 nl per oocyte (5 ng cRNA) using a Nanoject injector (Drummond, USA). Standard two-electrode voltage-clamp measurements were performed at room temperature (22°C–24°C) 2–5 days following cRNA microinjection. Oocytes were placed into a 100 µl recording chamber and superfused with a modified ND96 solution (containing 0.1 mM CaCl_2_) using a fast perfusion system which operates under controlled N_2_ pressure allowing constant perfusion velocity of 3.9–4.2 ml/min. The exchange of solutions was performed by computer-controlled pinch valves (ALA-VM8, ALA Scientific Instruments). A home made manifold having virtually no void volume and very narrow connecting tubes prevented backward flow upon valve switch. The bath solution was completely replaced within 1.5 seconds, allowing a solution exchange time of about 25 ms around the oocyte. Whole-cell currents were recorded using a GeneClamp 500 amplifier (Axon Instruments). Stimulation of the preparation, and data acquisition were performed using the pCLAMP 6.02 software and a 586 personal computer interfaced with a Digidata 1200 interface (Axon Instruments). Glass microelectrodes (A–M systems, Inc) were filled with 3 M KCl and had tip resistances of 0.2–0.5 MΩ. Current signals were digitized at 1 kHz and low pass filtered at 0.2 kHz. Errors introduced by the series resistance of the oocytes were not corrected and were minimized by keeping expression of the currents below 10 µA.

#### S4 mutagenesis, CHO cell culture and transfection

A pcDNA3-based vector encoding human KCNQ1 served as a template to generate the S4 mutants using standard PCR techniques. The PCR-amplified regions of all mutants were verified by DNA sequencing. CHO cells were plated on poly-D-lysine-coated glass coverslips and grown in Dulbecco's modified Eagle's medium supplemented with 2 mM glutamine, 10% fetal calf serum and antibiotics. Transfections were carried out with pIRES-CD8 (0.5 µg) as a marker for transfection and with KCNQ1 wild-type (WT) and mutants (0.5 µg), using FuGENE (Roche Diagnostics) [26].

#### Patch-clamp Electrophysiology

Recordings were performed 40–48 h following transfection, using the standard whole-cell patch-clamp technique. Signals were amplified using an Axopatch 200B patch-clamp amplifier, sampled at 2 kHz and filtered at 800 Hz. Data were acquired using pClamp 8.1 software with a DigiData 1322A interface (Axon Instruments). The patch pipettes were pulled from borosilicate glass (Warner Instrument. Corp) with a resistance of 2–5 MΩ and were filled with (in mM): 130 KCl, 1 MgCl2, 5 K_2_ATP, 5 EGTA, 10 HEPES, adjusted with KOH at pH 7.4 (290 mOsm). The external solution contained (in mM): 140 NaCl, 4 KCl, 1.8 CaCl2, 1.2 MgCl2, 11 glucose, 5.5 HEPES, adjusted with NaOH at pH 7.4 (310 mOsm). Channel expression into Xenopus oocytes and two-electrode voltage-clamp recordings were performed as described [27].

#### Data analysis

Conductance (G) obtained from tail current amplitudes or from steady-state currents (when deactivation was very fast) was calculated by the following equation G = I/(V−V_rev_) where the calculated reversal potential V_rev_ was −90 mV. G was then, normalized to the maximal conductance value, G_max_. Activation curves were fitted by a single Boltzmann distribution G/G_max_ = 1/{1+exp[(V_50_−V)/s], where V_50_ is the voltage at which the current is half-activated and s is the slope factor. For the thermodynamic analysis, the difference in Gibbs free energy between closed and open states at 0 mV (ΔG_0_) was calculated according to ΔG_0_ = 0.2389 zFV_50_. The change in free energy difference between WT KCNQ1 and the mutant was computed by ΔΔG_0_ = ΔG_0_^mut^−ΔG_0_^wt^. To take into account the differences in side chain volume ∥Δvol∥ produced by the tryptophan mutations, ΔΔG_0_^c^ values were corrected according to the relative change in side chain volume ΔΔG_0_^c^ = ΔΔG_0_ [∥Δvol^av^/Δvol∥], with an average change vol^av^ = 68 Å^3^, as previously described [21]. All data were expressed as mean ± SEM. Statistically significant differences between paired groups were assessed by a two-tailed Student's t-test. Statistically significant differences between unpaired groups were assessed using one way ANOVA followed by Dunnett's Multiple Comparison Test.

#### Model building

The model of the pore and sensor domains of Kv1.7 was built as in Haitin et al. (this issue). The membrane-embedded segment of the KCNE1 auxiliary subunit was folded as a classical alpha helix. Then, this helix was docked to the open state by enabling sufficient proximity of KCNE1-Glu43 to Kv1.7-Ala226, so as to account for the findings of Nakajo and Kubo [19] who reported on the formation of an open-channel stabilizing disulfide bridge between cysteine residues introduced at the aforementioned positions. The lower part of the helix was placed as close as possible to the pore domain avoiding any clashes. This specific orientation could not be kept in the closed-channel conformation since the bottom (more intracellular) portion of KCNE1 severely clashed with the S1 segment of the adjacent subunit. Therefore, in the case of the closed-channel conformation, the bottom part of the KCNE1 helix was moved laterally, resulting in a tilt, to preserve the same distance from the pore domain, as modeled for the open state. The disposition of KCNE1 proposed here is in line with our current studies showing that the VSDs of two adjacent subunits get closer to one another during activation. The latter mechanism would predict drastic interference of KCNE1 to activation, which is indeed reflected by slow activation rates of Kv1.7 in the presence of KCNE1.
